# Electrostatic Similarities between Protein and Small Molecule Ligands Facilitate the Design of Protein-Protein Interaction Inhibitors

**DOI:** 10.1371/journal.pone.0075762

**Published:** 2013-10-10

**Authors:** Arnout Voet, Francois Berenger, Kam Y. J. Zhang

**Affiliations:** Zhang Initiative Research Unit, Institute Laboratories, RIKEN, Wako, Saitama, Japan; Griffith University, Australia

## Abstract

One of the underlying principles in drug discovery is that a biologically active compound is complimentary in shape and molecular recognition features to its receptor. This principle infers that molecules binding to the same receptor may share some common features. Here, we have investigated whether the electrostatic similarity can be used for the discovery of small molecule protein-protein interaction inhibitors (SMPPIIs). We have developed a method that can be used to evaluate the similarity of electrostatic potentials between small molecules and known protein ligands. This method was implemented in a software called EleKit. Analyses of all available (at the time of research) SMPPII structures indicate that SMPPIIs bear some similarities of electrostatic potential with the ligand proteins of the same receptor. This is especially true for the more polar SMPPIIs. Retrospective analysis of several successful SMPPIIs has shown the applicability of EleKit in the design of new SMPPIIs.

## Introduction

With the advent of the ‘omics’ era, it has become clear that most proteins do not act in solitude but depend on Protein-Protein Interactions (PPIs) to exert their biological function. It has been estimated that the number of PPIs in humans ranges from ∼130,000 [1] to ∼650,000 [2] and these PPIs are crucial for the regulation of many biological processes. PPIs are often involved in processes associated with diseases, therefore targeting PPIs with small molecule PPI inhibitors (SMPPIIs) opens a pipeline for the development of novel drug classes against a variety of diseases.

While many small molecule drugs targeting enzymes, nuclear receptors, ion channels and G-protein coupled receptors have been developed, the number of reported successes in the discovery of SMPPIIs remains fairly low. As a matter of fact, PPIs were once thought to be high hanging fruits for drug discovery [3]. PPIs were even considered to be undruggable, mostly because of their relative flat but extensive interfaces [4]. Though initially thought to be undruggable, an increasing number of SMPPIIs have been reported in recent years [5]. However, the number of deposited 3D SMPPII receptor complex structures remain far more limited than the number of reported successful cases. This hinders the understanding of their mechanism of action and chemical space properties [6]. Commonly used methods for screening are computational docking [7] and pharmacophore-based screening [8]. It was observed that the crucial interactions between a protein ligand and its protein receptor are often similar to those between the SMPPII and the protein receptor [9], [10]. Thus, the PPI interface can be used to create a pharmacophore query to screen for small molecule ligands [11], [12].

Another approach is to exploit the principle of electrostatic complementarity in molecular recognition. Next to steric complementarity, electrostatics are one of the main driving forces involved in molecular recognition [13]. Despite the complex biophysical nature of the electrostatic potential, calculations for macromolecular systems are nowadays tractable [14], [15].

Electrostatics are known to play a key role in protein-DNA [16], protein-protein [17] and protein-substrate [13] recognitions. Given the importance of electrostatics for the molecular recognition event, electrostatics have been used to study protein similarity [18]–[20] and the nature of protein-protein interactions [17], [21]–[24]. More specifically, the electrostatic complementarity between protein-protein interfaces has long been a subject of investigation [22], [23]. Using the correlation of electrostatic potentials as a quantitative measure, the electrostatic complementarity between PPI interfaces has been demonstrated [17], [24]. Other studies focused on the conservation of the electrostatic potentials through evolution [25] and its role in molecular association kinetics [26].

It is generally accepted that there is a high degree of complementarity in shape and electrostatics between a ligand and its receptor. This implies that molecules with similar shape and electrostatic properties may bind to the same receptor. This principle has been used to identify small molecule inhibitors similar to natural substrates or known inhibitors by screening for compounds with similar shape, volume and electrostatics [27]–[30].

An SMPPII cannot occupy the same shape and volume as its much bigger protein-ligand counterpart. However, it can still be assumed that there is some local electrostatic potential similarity between an SMPPII and a ligand protein, since they recognize the same binding site on the receptor. A recent example of the usefulness of taking electrostatic potential similarity into account while designing an SMPPII can be found in the work of Cavalluzo *et al.* [31], where an SMPPII was designed *de novo* by including electrostatic similarity. This success has motivated our effort to systematically investigate the complementarity in electrostatic potential between small molecules and protein ligands binding to the same protein receptor, and its potential use to assist in the rational design of SMPPIIs. For this purpose, a tool named EleKit was developed.

## Methods

To compute the partial charges and electrostatic potentials, EleKit builds upon PDB2PQR [32] and APBS [15]. EleKit requires two sets of complex structures in order to calculate the electrostatic similarity between a protein ligand and a small molecule ligand: (i) the PPI complex of the protein-ligand (L_P_) with the protein-receptor (R_P_) and (ii) a small molecule ligand (L_SM_) in its predicted or experimentally determined conformation on the protein-receptor (R_P_).

**Figure 1:**
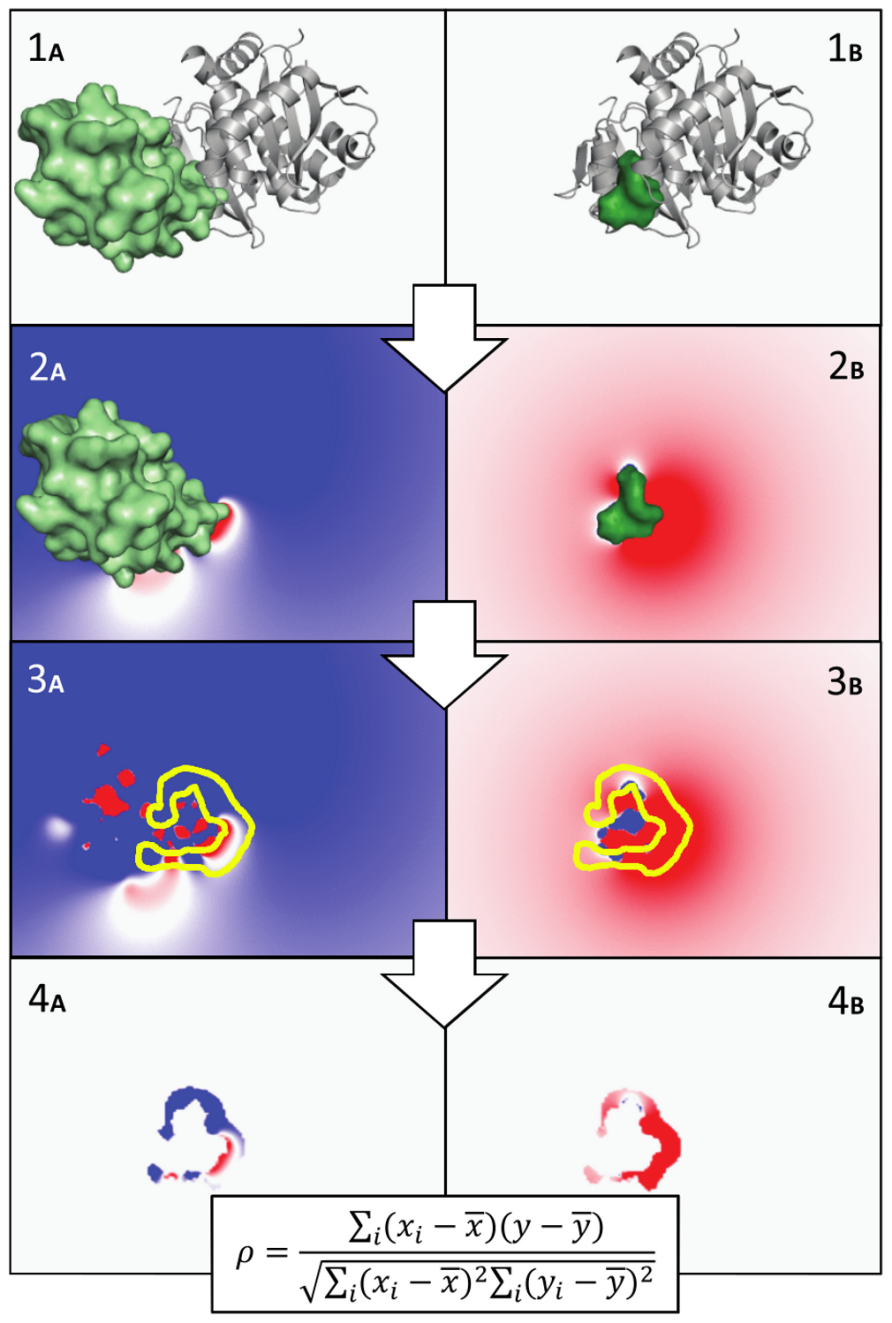
Overview of EleKit applied to PDB codes 2B4J (1_A_) and 3LPU (1_B_). The ligand protein (L_P_) is shown as a green surface in 1_A_ and 2_A_. The ligand small molecule (L_SM_) is shown as a smaller green surface in 1_B_ and 2_B_. The receptor protein (R_P_) is shown as a gray cartoon in 1_A_ and 1_B_. and are placed on (1_A_ and 1_B_). The electrostatic potentials of and are calculated and stored in distinct grids (2_A_ and 2_B_). Then, a mask is created to select the solvent region near the interface (3_A_ and 3_B_). Finally, the similarity between electrostatic potentials of and over this region is calculated using the Spearman rank correlation coefficient (4_A_ and 4_B_).

**Figure 2:**
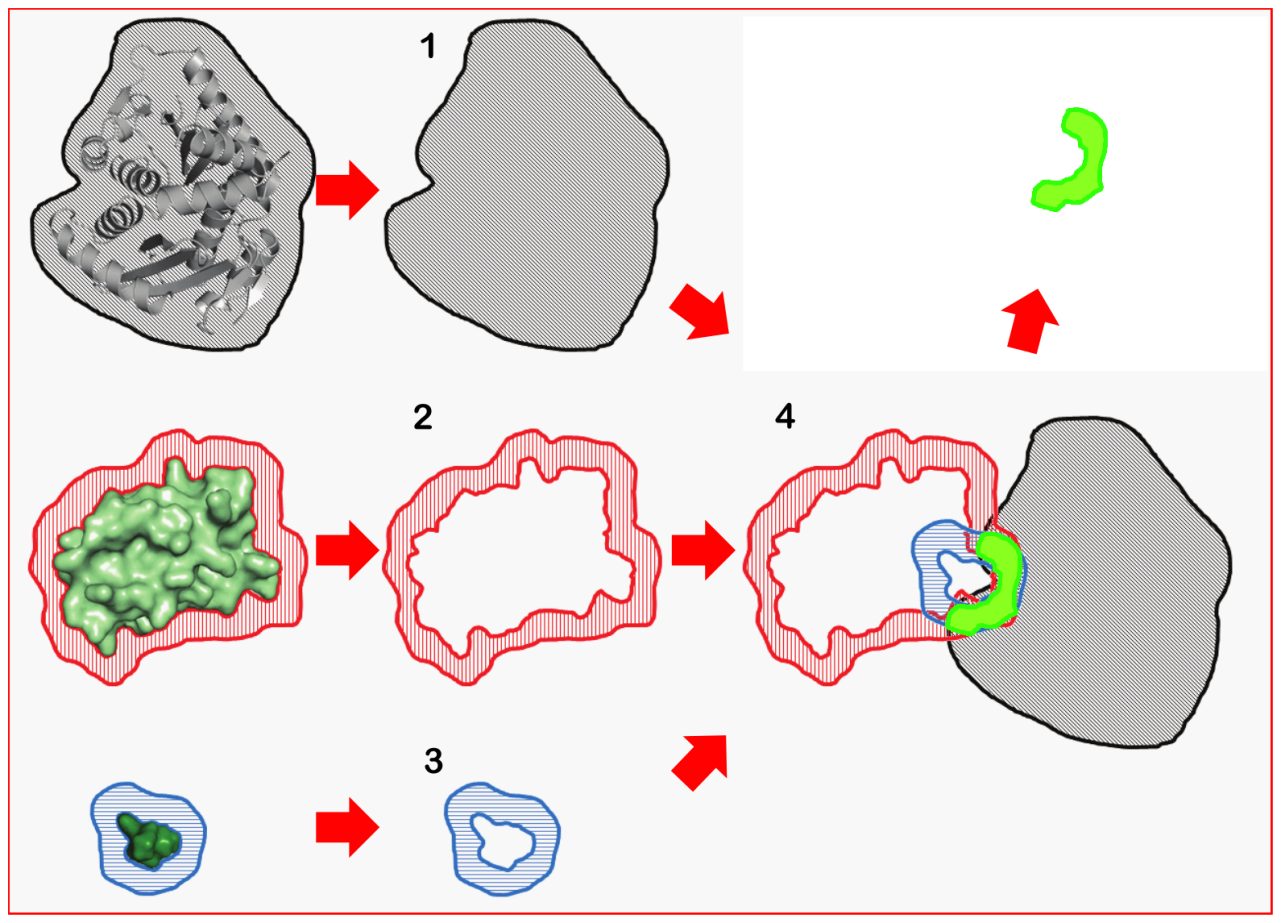
Overview of the bit-mask construction in EleKit. (1) a near-or-inside mask of R_P_ is created, (2) a near-but-not-inside mask of L_P_ is created, (3) a near-but-not-inside mask of L_SM_ is created, (4) the logical conjunction of the three masks is used to select points to correlate from the electrostatic potentials of L_P_ and L_SM_.

The EleKit method is shown schematically in figure 1. First, the electrostatic potentials around and are computed using APBS (parameters listed in table 1) and stored in 3D grids. Since only the area where and intersect is most likely to be relevant for molecular recognition, a bit mask is created on the electrostatic potential grids (figure 2). The goal of this mask is to take into account only those points in space that are not only in the solvent region around and but also near the interface atoms of R_P_. To create this mask, a distance cutoff is needed. This distance is used when dilating (a morphological mathematical operation) the molecular surface. Based on the hydrogen bond length (∼2.5Å) and the facts that enough points are needed for correlation and that the local similarity is our focus, a cutoff value ranging from 1.4 Å to 3.5 Å seems reasonable. All experiments reported in this study were performed with an intermediate cutoff value of 2.0 Å. Using 3.0 Å or 4.0 Å would have very little impact on the results (data not shown). Finally, the similarity between electrostatic potentials of and is assessed by correlating values at the grid points within the mask using the Spearman rank-order correlation coefficient (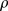). Additional similarity scores (Carbo index [33], Hodgkin index [34], Pearson's *r* and a Tanimoto score) are also calculated.

**Table 1:** APBS commands used for a protein.

read	
	mol pqr ligandprotein.pqr
end	
elec	
	mg-auto
	dime 161 193 161
	cglen 105.5210 127.4643 91.1458
	fglen 82.0712 94.9790 73.6152
	cgcent -6.603267 -6.904766 -18.393622
	fgcent -6.603267 -6.904766 -18.393622
	mol 1
	lpbe
	bcfl sdh
	pdie 2.0000
	sdie 78.5400
	srfm smol
	chgm spl2
	sdens 10.00
	srad 1.40
	swin 0.30
	temp 298.15
	calcenergy no
	calcforce no
	write smol dx ligandprotein.ms
	write pot dx ligandprotein
end	
quit	

For a small molecule, the input and output file names would differ. Parameters were determined with pdb2pqr.py. Grid-related parameters vary upon each case (dime, cglen, fglen, cgcent and fgcent).

EleKit is written in OCaml [35] (http://biocaml.org) and computations are parallelized by the Parmap library [36]. Experiments were run on Linux computing nodes with 2.4GHz Intel Xeon processors. EleKit takes between ten seconds to two minutes per ligand molecule and can parallelize the computation of several ligands when run on a multi-core machine. EleKit is released as open source and available from the authors' website http://www.riken.jp/zhangiru/software.html.

## Results and Discussion

The EleKit method was applied to analyze previously reported cases of SMPPIIs, for which accurate structures of the PPI as well as the SMPPII receptor complex are available in the PDB (table 2). Additionally, the SMPPIIs are required to bind in the PPI interface, allowing for a substantial overlap between the protein ligand and the SMPPII and thus excluding allosteric inhibition mechanisms.

**Table 2:** Analysis of SMPPII electrostatic mimicry.

PPI target	SMPPI complex	#points	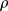	*r*	t_2_
	(observations)				
HDM2:p53	1rv1	5515	0.275	0.263	0.816
(1ycr)	1t4e (g)	5281	0.433	0.355	0.757
	1ttv	4862	-0.152	-0.123	0.588
	3jkz	4384	0.200	0.018	0.566
	3lbj (g)	5159	0.353	0.190	0.611
	3lbk (g)	4681	0.316	0.209	0.883
	3lbl	4129	0.131	-0.015	0.672
	3tu1 (g)	4492	0.589	0.329	0.764
	4dij	4758	0.239	0.111	0.619
IL2:IL2R	1m48 (g)	3186	0.315	0.276	0.658
(1z92)	1m49 (g)	3523	0.758	0.654	0.737
	1py2	3231	-0.011	0.095	0.583
	1pw6 (g)	4119	0.653	0.574	0.678
	1qvn	3819	0.093	0.098	0.626
Integrase:LEDGF/p75	3lpt (g)	3653	0.468	0.437	0.205
(2b4j)	3lpu (g)	3489	0.460	0.464	0.220
	4dmn (g)	3880	0.471	0.534	0.229
	4e1m (g)	3612	0.609	0.601	0.228
	4e1n (g)	3486	0.615	0.591	0.267
Integrin:Fibrinogen	2vdm (g)	6471	0.731	0.751	0.617
(2vdo)	2vc2 (g)	6469	0.736	0.720	0.614
VHL:HIF1 (1lqb)	3zrc (g)	4135	0.123	0.127	0.062
XIAP:smac	2i3i (g)	5979	0.535	0.501	0.650
(1g3f)	1tft (g)	5853	0.377	0.409	0.525
	3eyl (g)	4377	0.321	0.375	0.506
	2jk7 (g)	5446	0.464	0.447	0.631
	3clx	5570	0.055	0.127	0.431
	3cm7	6005	0.227	0.229	0.534
	3hl5	6044	0.263	0.302	0.484
	3mup (g)	6028	0.480	0.404	0.671
	3g76 (g)	5008	0.396	0.391	0.371
	3oz1	5340	0.191	0.184	0.580
ZipA:FtsZ	1s1s (w_i_)	4320	0.150	-0.061	0.704
(1f47)	1y2f (w_i_, g)	3878	0.365	0.173	0.604
	1y2g (w_i_)	3809	-0.318	-0.147	0.335
	1s1j (w_i_, g)	2691	0.331	0.088	0.465

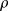
: Spearman rank correlation coefficient; r: Pearson linear correlation coefficient; t2: a Tanimoto score (positive or negative electrostatic potential); observations: **g** =  good (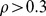), **w_i_** =  weak inhibitor (potency <100 µM in the literature).

The approach used in EleKit to perform comparison of electrostatic potentials resembles what has been done previously on proteins [18]–[21]. Analysis of Electrostatic Similarities of Proteins (AESOP) [20], the method of Dlugosz *et al.* [19] and Protein Interaction Property Similarity Analysis (PIPSA) [18], [37] also use APBS as their electrostatic computation engine. PIPSA can also use University of Houston Brownian Dynamics [38] (UHBD). While EleKit relies on the Spearman rank-order correlation coefficient (as McCoy *et al.* [17]), PIPSA uses the Hodgkin index [34] to numerically assess the similarity of electrostatic potentials. AESOP uses the Average Normalized Difference [39]. The method of Dlugosz *et al.* [19] approximates the electrostatic potential with spherical harmonics and uses a similarity index specifically designed to compare the obtained rotation-invariant descriptors. EleKit, similarly to several other methods [18], [19], [37], uses boolean masks to select a region over which electrostatic potentials are compared. All methods vary in the way masks are constructed.

### Analysis with EleKit

Electrostatic similarity analysis for these different SMPPII-related structures indicate that several exhibit correlation. In general, correlation between electrostatic potentials of SMPPIIs and electrostatic potentials of the respective ligand proteins are observed (table 2). This is especially true for the SMPPIIs targeting the HDM2:p53, HIV-1 Integrase:LEDGF/p75, Integrin:Fibrinogen, IL2:IL2R and XIAP:smac interactions. The highest similarity between a protein ligand and a small molecule ligand can be observed in the HIV-1 Integrase:LEDGF/p75 and the Integrin:Fibrinogen interactions and their respective inhibitors. In these cases, 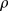 is on average ∼0.52 and ∼0.73 respectively (table 2). The origin of these classes of SMPPIIs can be traced back to pharmacophore based discovery of lead compounds designed to mimic the interactions observed at the PPI interface [40], [41].

For the inhibitors of the HDM2:p53 interaction, the majority of the inhibitors exhibit electrostatic potential similarity. However, a few show low correlations (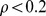) and in one case even some anti-correlation (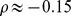). Interestingly, the Tanimoto score shows similarity in all HDM2:p53 cases. The electrostatic potentials between inhibitors and protein ligands in ZipA:FtsZ and VHL:HIF1 still correlate although less strongly than in other cases. These inhibitors are observed to be less active when tested. For inhibitors targeting the XIAP:smac interaction, which originated from peptidomimetic design, some compounds exhibit lower similarity than expected. This can be explained by the divergence of conformations of the receptor protein, since the XIAP:smac complex was solved by NMR while the structures of XIAP bound to inhibitors were solved by X-ray crystallography. The PPI complex solved by NMR spectroscopy are more difficult to superpose onto the crystal structure conformation obtained for the SMPPII complex. The inhibitors of the IL2:IL2R interaction are well known for binding to the IL2R interface by causing a rotameric change of a phenyl alanine creating a binding pocket [42]. In this case, the PPI interface is only partially covered in a hydrophobic area caused by the induced fit. However, the observed similarity between the ligand protein and the inhibitor mainly originates from the mimicry of the arginine guanidinium group, which is not influenced by conformational changes or induced fit.

There are no significant electrostatic correlations found in the cases of the inhibitors of the Bcl2 family of proteins, the TNF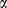 trimerization and the HPV polymerase. A careful analysis of the structures of these molecules revealed that the SMPPII in these cases is bound after a major reorganization of the receptor protein surface at the PPI interface. For the SMPPIIs bound to the Bcl2 family proteins, there is a major induced fit not only involving side chain atoms, but also including a rearrangement of a single helix, in order to comfortably fit the SMPPII inside the same cleft that was originally occupied by a full and more bulky 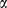-helical protein ligand. The inhibitors of the TNF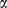 and HPV polymerase bind in a pocket at the PPI interface created by the assumption of different side chain orientations with more open conformations. Furthermore, the SMPPIIs that break the E1:E2 interaction of the HPV polymerase act as a dimer. In these cases, the SMPPIIs do not act by mimicking and competing with the ligand protein and no similarity of electrostatic potentials is observed.

EleKit is able to assess electrostatic potential similarity by a variety of measures including 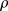, *r* and a Tanimoto score (table 2). Overall, relying on 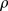 over *r* is preferred as it is more robust and does not suffer from uncertainties in interpreting the significance of the observed correlations [43]. The Tanimoto score, which is based on binning the electrostatic potential values as positive or negative does not seem to work in cases where the overall charge of the protein ligand is significantly different from that of the small molecule. An example is the case of the inhibitors of the Integrase:LEDGF/p75 interaction, where the protein ligand has a high positive net charge while the SMPPII has one negatively charged group. The Tanimoto scores are significantly affected and tend to be low. This problem is not observed when using correlation scores. The local shape similarity between the protein ligand and an SMPPII in EleKit is reflected by the number of electrostatic potential grid points being correlated. The more locally similar in shape a ligand protein is to a given SMPPI, the more grid-points will remain in the final mask.

### Application of EleKit

To test the utility of EleKit for the post-filtering of results from virtual screening such as docking, four different cases where strong electrostatic similarities between SMPIIs and protein ligands have been observed were selected for further analysis. In each case, a set of 100 diverse decoy compounds that bear similar chemical properties as the active SMPPII were extracted from the purchasable subset of the ZINC database [44] using the procedure described by Huang *et al.* [45], [46] to create the Directory of Useful Decoys (DUD).

To position these decoy compounds inside the receptor protein, as if it were a virtual screening experiment, molecular docking was performed using the Molecular Operating Environment (MOE) with the London dG score [47], [48]. The receptor protein structure was extracted from the coordinates of the respective PPI complex structure and optimized using the Protonate3D functionality from MOE [47]. Similarly, the active compounds were also docked inside their respective receptor structures. For each compound, the top scoring docked pose was retained and the electrostatic potential similarity with the ligand protein was calculated.

**Figure 3:**
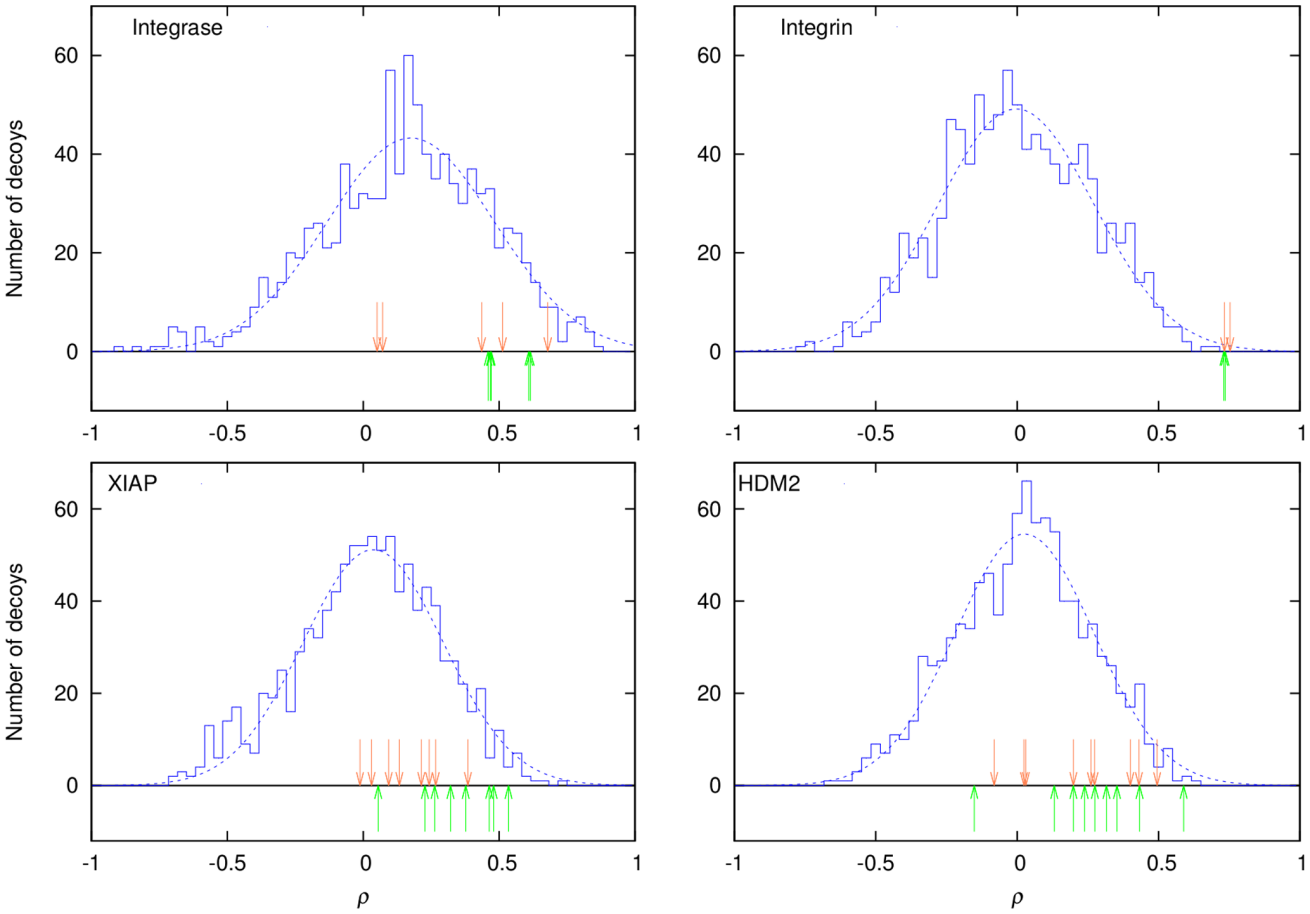
Distribution of Spearman scores (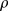) for active ligands among decoys. EleKit Spearman scores for a population of ligand decoys are shown as a blue histogram. The active ligands at their best docked pose in terms of RMSD to the crystallized active ligand are shown as orange arrows and the crystallized active ligands as green arrows. The dotted line is a Gaussian fitted to the decoys' histogram.

As expected, EleKit scores (based on a ligand protein and a ligand small molecule pair) are uncorrelated to docking scores (based on a ligand small molecule protein complex). The Spearman correlation between docking scores and EleKit scores fall in the range [−0.1;0.1] in every case (data not shown). In all four cases, the Spearman rank correlation coefficient for the decoys follows a Gaussian distribution (figure 3). The mean of the distribution is situated at 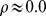 with an exception for HIV-1 Integrase:LEDGF/p75 (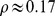). In this case, the active SMPPIIs are all mainly hydrophobic in nature except for one acid functional group, thus all decoys also bear a similar group. During the placement of those decoys into the hydrophobic pocket, this functional group has a high likelihood of adopting a similar arrangement to the active compounds, creating a higher correlating tendency and shifting the mean 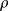. This distribution is independent from the scoring function as revealed by the low *R* ^2^ values for the correlation between London dG and 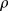. The highest observed value was 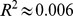 for the decoys of the Integrin:Fibrinogen interaction.

When SMPPIIs in their experimentally determined binding modes and with their best docked conformations are considered, a positive 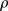 is always observed (figure 3). Only in the case of HDM2:p53, one of the nine active molecules has a negative 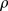. Furthermore, in the cases of the Integrin:Fibrinogen and Integrase:LEDGF/p75, their 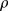 values reside on the even higher end of the distribution. In general, a cut-off value of at least 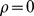 can be suggested as it would rightfully discard approximately half of the decoys. Such filtering using 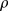 would only remove one inhibitor of the HDM2:p53 interaction, based on experimentally determined poses. However, the electrostatic similarity for docking results is influenced by the correctness of the predicted binding mode. In the Integrase case, the docking algorithm was unable to identify the correct binding mode within it's top solution for two compounds (the two orange arrows nearest to 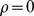 in the Integrase plot of figure 3). For one of these compounds, this can be explained by the small size of the molecule (PDB: 3lpt) and its lack of an important hydrophobic group, which leaves a huge hydrophobic cavity in the protein receptor unoccupied. However, the docking algorithm forced this small inhibitor to fill the unoccupied hydrophobic cavity resulting in a wrong predicted binding mode for this inhibitor. The second compound (PDB: 4e1n) has a significantly larger substituent group and would require a minor induced fit to bind correctly. The conformational difference of the receptor protein between its ligand protein and ligand small molecule bound forms can be problematic. In the case of the XIAP:smac inhibitors, this conformational difference exists since the structure of the PPI complex was determined using NMR spectroscopy and the structures of the SMPII complexes were determined by X-ray crystallography. The hydrophobic nature of the receptor protein can be a challenge. In the HDM2:p53 interaction, only a limited number of polar interactions that might help orienting the molecules in the right binding mode are present in the pocket.

**Figure 4:**
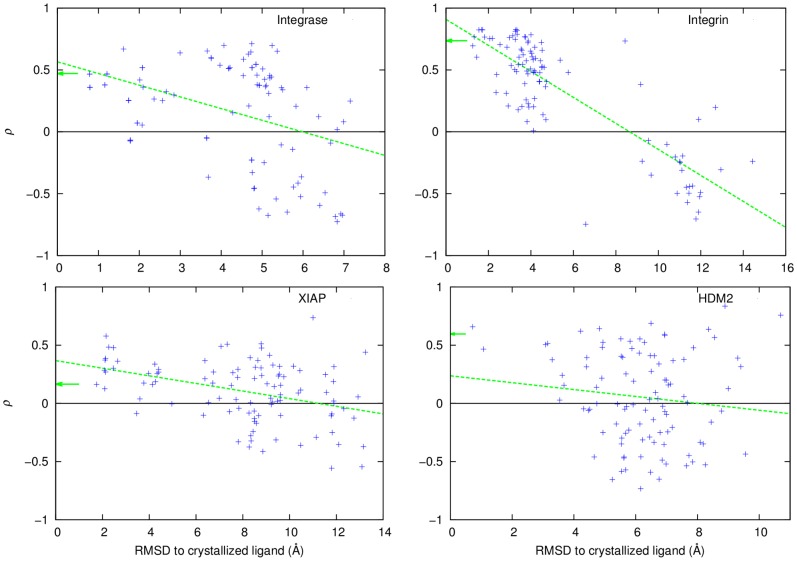
EleKit Spearman scores (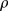) versus RMSD to crystal structure for computationally docked poses of known active ligands. Each green arrow indicates the Spearman score for the known active ligand as seen in the crystal structure. The green dotted line was obtained using gnuplot's fit command (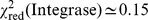, 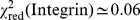, 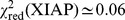, 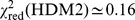).

**Figure 5:**
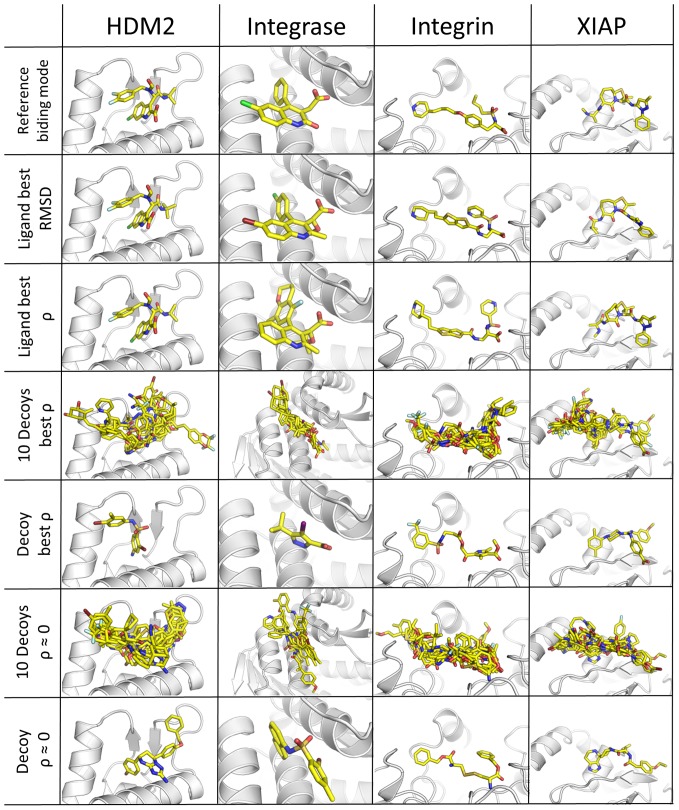
Analysis of docking poses. Comparison of binding modes predicted by docking for known active ligands and decoys to the reference ligands binding mode as experimentally determined. The best poses based on RMSD to the crystal structure, the pose with best 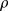 (ligand and decoys) and poses with 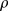 near zero (decoys) are shown. These binding modes indicate that the binding mode of the docked compounds are similar to the binding observed in X-ray or NMR structures. Despite the decoy compounds have binding modes in which their (a)polar contacts are similar to those of the active ligands, their electrostatic similarity with the ligand protein is different.

An overall analysis of the docked conformation revealed that in every case the docking algorithm was able to reproduce binding modes of the active compounds in agreement with the crystallographically determined binding modes. In the four receptors examined in details (Integrase, Integrin, XIAP, HDM2), computational docking was able to place the active ligands in binding modes almost identical to those determined crystallographically (RMSD less than 1 or 2 Å, figure 4). The higher 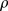 even corresponds to binding modes that are closer in RMSD (Fconv [49] was used to compute RMSD for small molecules) to the experimentally determined poses. Similarly, the decoy compounds were docked within the right binding pocket making similar contacts with the receptor protein as the active compounds (figure 5), therefore validating the suitability of the docking simulations. Despite the decoy compounds made similar contacts compared to the binding modes of the active ligands, it is clear that the electrostatic similarity of the decoy compounds with the ligand protein has a normal distribution, with its mean 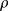 around 0. The ligands presented similar chemical groups in similar places driven by the complementarity of polar interactions in the pocket in a majority of the cases. The sole exception is found in the case of the HDM2/p53 that is hallmarked by a mainly apolar interface. Nevertheless, the apolar functions of the decoys and active ligands overlap in the binding mode.

The further away from the crystallographic pose the docked ligand is, the lower the Spearman rank correlation becomes. As a remark, the Receiver Operating Characteristic (ROC) analysis is typically used to assess the predictive and enrichment power of a method. But due to the lack of a significant number of active SMPPIIs for which structural information is available for a single target, this type of analysis could not be performed.

The development of EleKit was inspired by the computational work on electrostatic complementarity at protein-protein interfaces by McCoy *et al.* [17]. But EleKit bears salient differences with this former study. Whereas McCoy *et al.* studied the complementarity of protein-protein interfaces, EleKit measures the local similarity between one ligand protein and small molecules targeting the same receptor interface. McCoy *et al.* measured the correlation of electrostatic potentials at molecular surface points while EleKit works on a 3D volume in the solvent region near the binding interface. There are some significant prior works that compare electrostatic potentials and other molecular interaction fields for proteins only [18]–[21], [37], [50].

EleKit was also inspired by the commercial tools EON [27]–[29] and ElectroShape [30]. In effect, these tools can assess similarity of electrostatic potentials. However, they work exclusively on small molecules. Thus, they can identify compounds similar to a known small molecule inhibitor, but this is not applicable when searching for a first-in-class SMPPII [31].

## Conclusions

We have developed a method (EleKit) to investigate the similarity between protein ligands and their respective SMPPIIs. Our analysis of available SMPPII structures indicates that in cases where SMPPIIs bind without induced fit, there is similarity of electrostatic potentials at the interacting interface between a protein ligand and a small molecule inhibitor. This insight can be applied to post-filter virtual screening results to remove unpromising compounds and thus has some potential for the rational design of novel SMPPIIs.
